# Half-Sandwich Ru(II) and Os(II) Bathophenanthroline Complexes Containing a Releasable Dichloroacetato Ligand

**DOI:** 10.3390/molecules23020420

**Published:** 2018-02-14

**Authors:** Pavel Štarha, Zdeněk Trávníček, Ján Vančo, Zdeněk Dvořák

**Affiliations:** 1 Department of Inorganic Chemistry & Regional Centre of Advanced Technologies and Materials, Faculty of Science, Palacký University in Olomouc, 17. listopadu 12, 771 46 Olomouc, Czech Republic; pavel.starha@upol.cz (P.Š.); jan.vanco@upol.cz (J.V.); 2 Department of Cell Biology and Genetics & Regional Centre of Advanced Technologies and Materials, Faculty of Science, Palacký University in Olomouc, Šlechtitelů 27, 783 71 Olomouc, Czech Republic; zdenek.dvorak@upol.cz

**Keywords:** ruthenium, osmium, half-sandwich, dichloroacetate(1–), cytotoxicity, flow cytometry

## Abstract

We report on the preparation and thorough characterization of cytotoxic half-sandwich complexes [Ru(η^6^-*p*cym)(bphen)(dca)]PF_6_ (**Ru**-**dca**) and [Os(η^6^-*p*cym)(bphen)(dca)]PF_6_ (**Os**-**dca**) containing dichloroacetate(1–) (dca) as the releasable *O*-donor ligand bearing its own cytotoxicity; *p*cym = 1-methyl-4-(propan-2-yl)benzene (*p*-cymene), bphen = 4,7-diphenyl-1,10-phenanthroline (bathophenanthroline). Complexes **Ru**-**dca** and **Os-dca** hydrolyzed in the water-containing media, which led to the dca ligand release (supported by ^1^H NMR and electrospray ionization mass spectra). Mass spectrometry studies revealed that complexes **Ru-dca** and **Os-dca** do not interact covalently with the model proteins cytochrome c and lysozyme. Both complexes exhibited slightly higher in vitro cytotoxicity (IC_50_ = 3.5 μM for **Ru-dca**, and 2.6 μM for **Os-dca**) against the A2780 human ovarian carcinoma cells than *cisplatin* (IC_50_ = 5.9 μM), while their toxicity on the healthy human hepatocytes was found to be IC_50_ = 19.1 μM for **Ru-dca** and IC_50_ = 19.7 μM for **Os-dca**. Despite comparable cytotoxicity of complexes **Ru-dca** and **Os-dca**, both the complexes modified the cell cycle, mitochondrial membrane potential, and mitochondrial cytochrome c release by a different way, as revealed by flow cytometry experiments. The obtained results point out the different mechanisms of action between the complexes.

## 1. Introduction

Platinum(II) complexes *cisplatin*, *carboplatin,* and *oxaliplatin* represent the worldwide used metal-based anticancer chemotherapeutics [1]. It is well known that their application in clinics is connected with multiple drawbacks including nephrotoxicity, myelosuppression, and resistance of many tumors. Various strategies can be employed to improve the potency of the conventional platinum-based drugs and to circumvent the mentioned drawbacks [2,3]. One of them is based on the chemical modification of platinum(II) drugs, using their oxidation to Pt(IV) and coordination of bioactive ligands to the axial sites [4,5,6,7]. The first Pt(IV) complex of this type, *cis*,*cis*,*trans*-[Pt(NH_3_)_2_Cl_2_(dca)_2_] (*mitaplatin*) contains the orphan drug dichloroacetate (dca) coordinated to *cisplatin* [4]. The biological impact of *mitaplatin* is dual and affects both nuclear DNA (*cisplatin*) and mitochondria (dca) [4].

Osmium and especially ruthenium complexes have been intensively studied for their anticancer activity [8,9]. They act through different mechanisms of action than platinum-based drugs, resulting in different cytotoxicity profiles and ability to overcome both the intrinsic and acquired resistance of various tumors against platinum-based drugs. Importantly, several ruthenium complexes have entered the clinical trials as prospective non-platinum anticancer drugs [8,10]. Among the numerous types of anticancer ruthenium and osmium complexes, the half-sandwich complexes of the general formula [M(η^6^-ar)(XY)Z]^0/+^ represent a structural type showing the encouraging biological properties [11,12]; ar = six-membered arene ligand (e.g., *p*-cymene, *p*cym), XY = bidentate-coordinated ligand, Z = monodentate (typically halogenido) ligand. The mechanism of action is most likely based on a modulation of the redox status of the treated cancer cells [13,14]. Based on the extensive literature research, it is quite surprising that to date only one work has been dealing with the studies of cytotoxicity of half-sandwich Ru(II) or Os(II) complex containing the monodentate releasable bioactive ligand (in particular deprotonated ethacrynic acid, a known inhibitor of glutathione *S*-transferase isoenzymes [15], instead of the chlorido one [16]. By adopting the same premise, that it might be of great interest to investigate an impact of another type of simple bioactive carboxylate on cytotoxicity of half-sandwich non-platinum complexes, and having in mind the considerable anticancer effect of above-mentioned *mitaplatin* [4], we chose dichloroacetate as a suitable monodentate bioactive ligand for this research. Sodium dichloroacetate is an approved drug for the treatment of lactic acidosis, with promising cytotoxic properties [17]. The cytotoxic action of dichloroacetate is connected with an inhibition of pyruvate dehydrogenase kinase (PDK) resulting in a Warburg effect reversion, damage to the mitochondria and induction of apoptosis [18,19].

**Figure 1:**
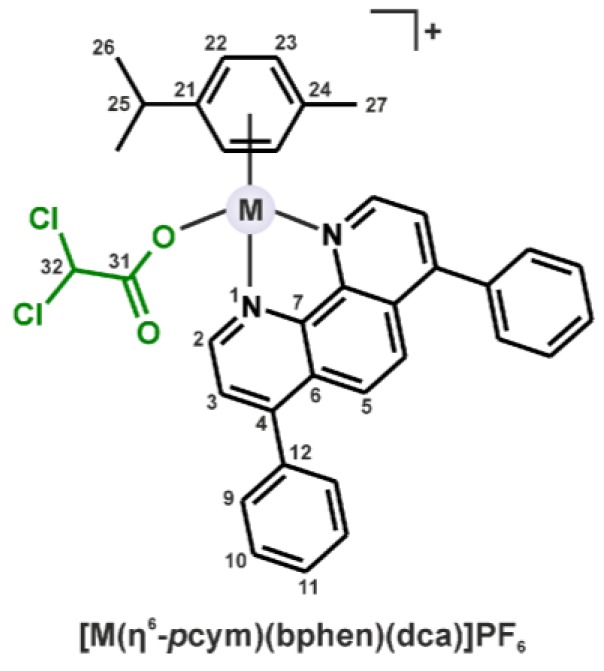
General structural formula of the [M(η^6^-*p*cym)(bphen)(dca)]PF_6_ complexes, where dca = dichloroacetate M = Ru for **Ru-dca** and Os for **Os-dca**), given with the atom numbering scheme.

Concerning the metal species used in this work, we utilized the formerly reported anticancer Ru(II) chlorido complex [Ru(η^6^-*p*cym)(bphen)Cl]PF_6_ (**Ru-Cl**) whose in vitro cytotoxicity against the A2780 cells (IC_50_ = 0.5 μM) was ca. 2-fold higher as compared with *cisplatin* (IC_50_ = 1.1 μM); bphen = 4,7-diphenyl-1,10-phenanthroline (bathophenanthroline) [20]. Herein, its new Os(II) analogue, [Os(η^6^-*p*cym)(bphen)Cl]PF_6_ (**Os-Cl**), was prepared and both Ru(II) and Os(II) chlorido complexes were modified by the replacement of the chlorido ligand by dichloroacetato ligand, providing complexes of the composition [Ru(η^6^-*p*cym)(bphen)(dca)]PF_6_ (**Ru-dca**) and [Os(η^6^-*p*cym)(bphen)(dca)]PF_6_ (**Os-dca**) (Figure 1). Complexes **Ru-dca** and **Os-dca** represent the first examples of the half-sandwich Ru(II) and Os(II) complexes containing dca.

## 2. Results and Discussion

### 2.1. Synthesis and Characterization

The chlorido complexes **Ru-Cl** (known from the literature [20]) and **Os-Cl** (newly prepared) were synthesized in the microwave reactor and subsequently used for the syntheses of the dichloroacetato complexes **Ru-dca** and **Os-dca** (Figure 1), representing the first examples of half-sandwich Ru(II) and Os(II) complexes containing the dca ligand. The complexes were characterized by elemental analysis (C, H, N), electrospray ionization (ESI+) mass spectrometry, and ^1^H and ^13^C NMR, and IR spectroscopies.

**Figure 2:**
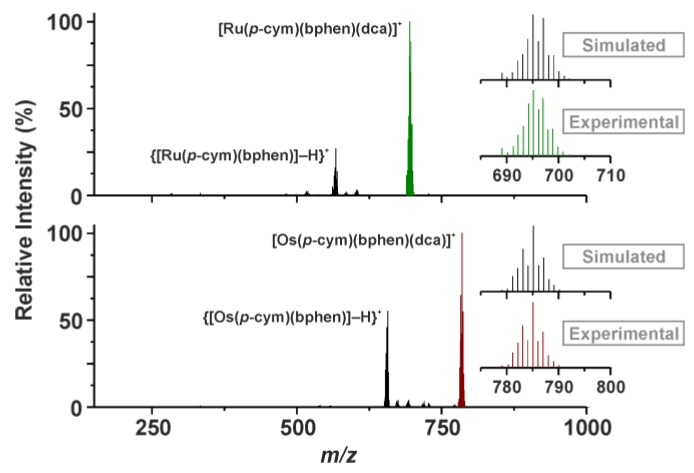
Electrospray ionization (ESI+) mass spectra of complexes **Ru-dca** (top) and **Os-dca** (bottom) given together with the details of the experimental and simulated peaks of the [M(*p*cym)(bphen)(dca)]^+^ species (M = Ru or Os).

The ESI+ mass spectra of complexes **Ru-dca** and **Os-dca** contained the peaks of 100% relative intensity, belonging to the complex [M(*p*cym)(bphen)(dca)]^+^ cations (Figure 2), which directly proved the formation of complexes **Ru-dca** and **Os-dca** from the **Ru-Cl** and **Os-Cl** starting compounds. Further, the peaks, assignable according their *m*/*z* values and isotopic distribution to the {[M(*p*cym)(bphen)]–H}^+^ species, were detected in the mass spectra of both complexes **Ru-dca** and **Os-dca** (Figure 2).

**Figure 3:**
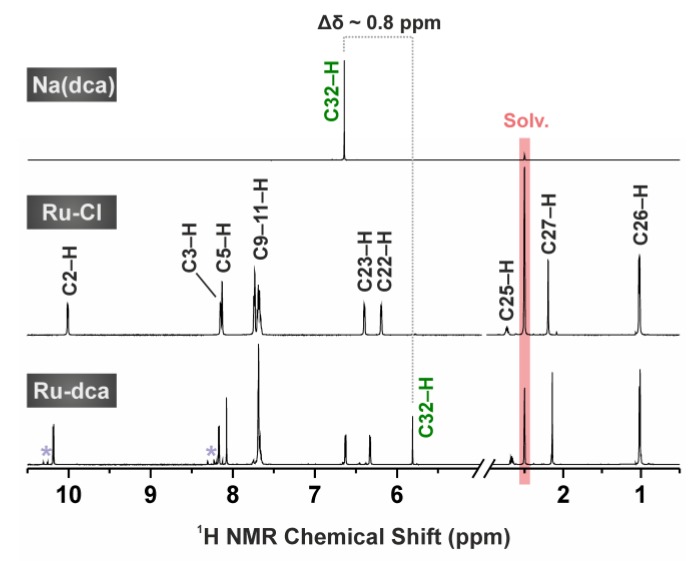
^1^H NMR spectra (DMSO-*d*_6_ solutions) of **Na(dca)** (top), complexes **Ru-Cl** (middle) and **Ru-dca** (bottom) given together with the assignment of the detected signals (see Figure 1 for the atom numbering scheme). Note: the impurity visible in the ^1^H NMR spectrum of complex **Ru-dca** can be assigned to the [Ru(η^6^-*p*cym)(bphen)(OH)]^+^ species (violet asterisks).

Although measured in different solvents, ^1^H NMR results of complex **Ru-Cl** (dissolved in DMSO-*d*_6_ or CDCl_3_ in this work; Figure 3 and Figure S1) were consistent with those previously reported for this complex (dissolved in acetone-*d*_6_) [20]. Complex **Os-Cl** had a similar pattern on the ^1^H NMR spectrum as the **Ru-Cl** complex, with the characteristic signals of the *p*cym aromatic hydrogens (C22–H and C23–H) shifted to the lower fields by ca. 0.2 ppm than for the **Ru-Cl** analogue. The replacement of the chlorido ligand of the precursors **Ru-Cl** and **Os-Cl** by the dca one became evident by a new ^1^H NMR signal (DMSO-*d*_6_ solutions) of the C32–H hydrogen atom of dca, detected at 5.81 ppm (for **Ru-dca**; Figure 3) and 5.86 ppm (for **Os-dca**). The C32–H signal of Na(dca) (measured in DMSO-*d*_6_) was found at 6.65 ppm, proving a coordination shift Δδ~0.8 ppm. Concerning the ^13^C NMR spectroscopy, the obtained spectra of the dca-complexes contained two new signals as compared with their chlorido analogues. These signals were detected at 168.3 ppm and 67.4 ppm (for **Ru-dca**), and at 168.1 ppm and 66.8 ppm (for **Os-dca**), and can be unambiguously assigned to the C31 and C32 atoms of the dca ligand. These signals were shifted by 2.4 ppm (for C31) and 1.5 ppm (for C32) for **Ru-dca**, and by 2.2 ppm (for C31) and 0.9 ppm (for C32) for **Os-dca**, as compared with Na(dca) (measured in the same solvent; δ (ppm) = 65.9 (C31), 165.9 (C32)).

The purity of complexes **Ru-dca** and **Os-dca** was >97% and >99%, respectively, based on the results of elemental analysis and ^1^H NMR spectroscopy (Figure 3). Regarding the stability in the used solvent (DMSO-*d*_6_), no new signals or chemical shift changes were observed in the ^1^H NMR spectra of the studied complexes **Ru-dca** and **Os-dca** even after 72 h of standing at ambient temperature.

### 2.2. ^1^H NMR Spectroscopy and ESI+ Mass Spectrometry Studies of Hydrolytic Stability

**Figure 4:**
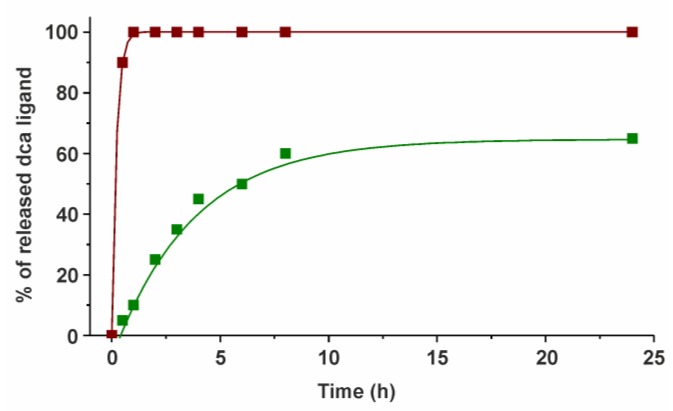
The results of the time-dependent ^1^H NMR studies showing the progress of the dca ligand release for complexes **Ru-dca** (brown solid squares) and **Os-dca** (green solid squares). Note: The lines are only guides for eyes.

Since the studied complexes **Ru-dca** and **Os-dca** were designed as prodrugs of two mechanistically independent cytotoxic species (i.e., metal-based species and released dca), the solution behavior in water-containing medium was studied by relevant techniques (NMR, ESI-MS) to show, whether the dca ligand releases under the used conditions. Both the complexes **Ru-dca** and **Os-dca** (1 mM concentration) hydrolyzed in the used water-containing mixture of solvents (20% MeOD-d_4_/80% D_2_O), where methanol ensured the sufficient solubility to reach relevant concentration (1 mM in this work) for ^1^H NMR experiments. Hydrolysis of the studied complexes is connected with the release of the dca ligand (Figure 4). A ^1^H NMR signal of C32–H of the coordinated dca ligand showed at 5.47 ppm for **Ru-dca** and at 5.48 ppm for **Os-dca**, while the released dca anion showed at 5.91 ppm in the ^1^H NMR spectra of both complexes (Figure S2). A position of this signal correlated well for the released dca and free dca (measured for Na(dca) in the same mixture of solvents) detected at 5.93 ppm. Complex **Ru-dca** hydrolyzed completely, while the hydrolysis rate was only ca. 65% for its Os(II) analogue after standing at room temperature for next 24 h (Figure 4). 

The ESI+ mass spectra of both complexes **Ru-dca** and **Os-dca** (10 µM concentration) contained the peaks assignable to the hydrolytic products of the parent complexes of the composition [M(pcym)(bphen)(OH)]^+^. As for **Ru-dca**, a ratio of intensities between the [Ru(pcym)(bphen)(dca)]^+^ (695.1 *m*/*z*) and hydrolyzed [Ru(pcym)(bphen)(OH)]^+^ (584.8 *m*/*z*) species equaled ca. 3:2 after standing at room temperature for 1 h, while a peak of the parent [Ru(pcym)(bphen)(dca)]^+^ species almost disappeared (<5% relative intensity) with ongoing hydrolysis after 24 h (Figure S3). In the case of complex **Os-dca**, the change of the ratio of intensities between the [Os(pcym)(bphen)(dca)]^+^ (785.3 *m*/*z*) and [Os(pcym)(bphen)(OH)]^+^ (675.3 *m*/*z*) species detected after 1 h (ca. 6:1) and 24 h (ca. 5:1) was negligible (Figure S3). Thus, the ESI+ mass spectrometry results, consistently with ^1^H NMR spectra (see above), provided proofs revealing a higher hydrolytic stability of the **Os-dca** complex as compared to its Ru(II) analogue.

### 2.3. Mass Spectrometry Studies of Interactions with Sulfur-Containing Biomolecules

To investigate better the behavior of the studied complexes in the presence of selected biomolecules, we performed the ESI+ MS experiments for the mixtures of complexes **Ru-dca** and **Os-dca** (10 µM final concentration) with the sulfur-containing biomolecules l–cysteine (CySH) and reduced glutathione (GSH) at their average levels in human plasma [21,22,23]. No peaks assignable to the covalent adducts with CySH or GSH were detected in the ESI+ mass spectra of the samples containing complex **Ru-dca** and the mixture of CySH and GSH after 24 h of incubation. However, the peaks assignable to the adducts of {[Ru(*p*cym)(bphen)]–H}^+^ with either two CyS (or one cystine (CySSCy); 807.2 *m*/*z*) or with CyS and GS (or their disulfide CySSG; 993.2 *m*/*z*) appeared in the ESI+ mass spectrum of the reaction system after 24 h of incubation (Figure S4). Moreover, a peak whose mass-to-charge ratio and isotopic pattern suggests the formation of adduct of {[Ru(*p*cym)(bphen)]+(HL^1^)–H}^+^ with deaminated cysteine (i.e., 3-sulfanylpropanoic acid; HL^1^), showed at 673.0 *m/z* in the ESI+ mass spectrum recorded after 24 h (Figure S4). Although the deamination reaction is not very common in connection with the electrospray ionization process, similar processes, such as the deamination of various anilines by the Ru-based catalyst [24] or cysteine deamination to β-mercaptopyruvate [25] were already described in the literature.

As for the **Os-dca** complex, the peaks of similar adducts described above for the the **Ru-dca** complex were also detected by ESI+ mass spectrometry in the mixture containing **Os-dca** complexes (i.e., {[Os(pcym)(bphen)]–H}^+^ with either two CyS or one CySSCy at 897.2 *m*/*z*, or with CyS and GS or disulfide CySSG at 1083.3 *m*/*z*), however their assignability is uncertain due to very low intensity. On the other hand, the ESI+ mass spectrometry experiments confirmed higher stability of the **Os-dca** complex in the presence of GSH. Specifically, the intensity ratio of [Os(pcym)(bphen)(dca)]^+^ and [Os(pcym)(bphen)(OH)]^+^ was ca. 8:1 after 1 h and ca. 6:1 after 24 h. Contrary to complex **Ru-dca**, no peak indicating the cysteine deamination was detected in the ESI+ mass spectra recorded on the mixture containing complex **Os-dca**.

### 2.4. Mass Spectrometry Studies of Interactions with Model Proteins 

The affinity of the complexes **Ru-dca** and **Os-dca** towards the model proteins cytochrome c (Cytc) and lysozyme (HEWL) was studied by ESI+ mass spectrometry. Both proteins were formerly reported as interacting with platinum-based drugs [26,27,28] as well as with similar half-sandwich Ru(II) [28,29,30,31,32,33] and Os(II) [34] complexes, thus they represent the suitable models to shed a light on whether the interactions with proteins should be taken into account as a possible aspect of mechanism of action/transportation/activation of the **Ru-dca** and **Os-dca** complexes. However, any peaks assignable to the adducts of the [M(pcym)(bphen)(dca)]^+^ complex cations or their fragments were not detected in the deconvoluted mass spectra of the mixtures of the studied dca-complexes with neither Cytc nor HEWL, even after 120 h after preparation of the reaction systems. The only adduct, visible after 24 h of incubation of Cytc with the studied complexes **Ru-dca** or **Os-dca** corresponded to the mass difference of 145 Da, assignable to the adduct of Cytc with the PF_6_^–^ ion (Figure S5). Such adducts appear commonly in the protein-metal complex systems containing the PF_6_^–^ anions studied by electrospray-ionization mass spectrometry as minor adducts, accompanying the major metalated ones [35,36]. However, in our case, we did not observe formation of such products.

### 2.5. In Vitro Cytotoxicity

The in vitro cytotoxicity of complexes **Ru-dca** and **Os-dca** was screened at the A2780 human ovarian carcinoma cell line. The dca-complexes were slightly more cytotoxic (IC_50_ = 3.5 ± 0.3 μM and 2.6 ± 0.4 μM for **Ru-dca**, and **Os-dca**, respectively) against the used A2780 cells than the clinically used platinum-based drug *cisplatin* (IC_50_ = 5.9 ± 1.2 μM); note*:* IC_50_ of Hdca equaled 5.0 mM with the same experimental conditions used. The relative activity (RA = IC_50_(*cisplatin*)/IC_50_(complex)) equaled ca. 1.7 and 2.3 for complexes **Ru-dca**, and **Os-dca**, respectively, which implied approximately 2-fold higher potency than determined for conventional *cisplatin*. Even if no direct comparison should be made between the IC_50_ values obtained by our experiments and the published results of cytotoxicity, it is important to note that the well-known Ru(II) complex KP1019, which have entered into the clinical trials, showed lower potency (IC_50_ = 77.8 μM) as compared to *cisplatin* (IC_50_ = 9.5 μM) at the A2780 cells (24 h exposure with no recovery, MTT assay), thus showing the RA value of ca. 0.1 [37]. Similarly, *mitaplatin*, a Pt(IV) complex containing the dca ligand (see Introduction), is also less effective (IC_50_ = 1.1 μM) than the reference drug *cisplatin* (IC_50_ = 0.6 μM) at the same A2780 cell line (72 h exposure with no recovery, MTT assay), resulting in the RA value of ca. 0.5 [4].

The cellular accumulation of the **Ru-dca** and **Os-dca** complexes equaled 366.1 ± 14.0, and 273.1 ± 6.4 fmol/10^6^ cells, respectively, indicating that the **Os-dca** complex was less accumulated within the cancer cells than its Ru(II) analogue. Taking into account the similar results of cytotoxicity of both complexes and on the other hand the differences in their cellular accumulation, these findings may indicate dissimilarity in molecular mechanisms of cytotoxicity of both complexes. In addition, both the dca-complexes exceeded the cell uptake level of their chlorido precursors (see Table 1). 

In vitro toxicity of the **Ru-dca** and **Os-dca** complexes was studied on the primary culture of human hepatocytes. The obtained results indicated that the toxic effect of complexes **Ru-dca** (IC_50_ = 19.1 μM) and **Os-dca** (IC_50_ = 19.7 μM) is lower than their cytotoxicity against the A2780 ovarian carcinoma cells.

### 2.6. Cell Cycle Analysis

**Figure 5:**
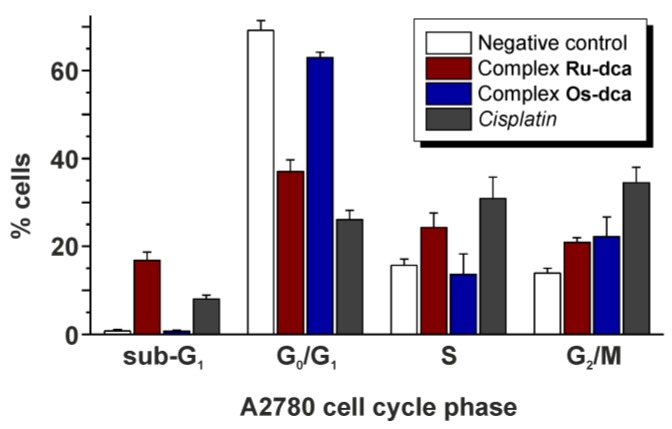
The A2780 cell cycle phase populations (%) after the 24 h treatment by the IC_50_ concentrations of complexes **Ru-dca** and **Os-dca**, given together with untreated cells (negative control) and the cells treated with platinum-based drug *cisplatin*. The data are given as arithmetic mean from three independent experiments.

The **Ru-dca** and **Os-dca** complexes modified the cell cycle of the A2780 human ovarian carcinoma cells differently, when applied at equipotent (IC_50_) concentration (Figure 5, Table 2). In particular, the **Ru-dca** complex strongly increased the sub-G_1_ cell population (16.8% of cells) as comparted to the **Os-dca** complex (0.7% of cells). The increase in the sub-G_1_ population is often connected with the appearance of late-apoptotic and necrotic cells. Simultaneously, the **Ru-dca** complex caused the decline in the G_0_/G_1_ cell cycle phase population (37.0% of cells) as compared with the negative control (69.2% of cells) as well as with complex the **Os-dca** complex (62.9% of cells). In the S-phase, the effect of the **Ru-dca** complex is much closer to that of *cisplatin* (24.3% vs. 30.9% of cells) as compared with the **Os-dca** complex (13.6% of cells). In the G_2_/M cell phase, both the complexes showed the similar effect (20.9% vs. 22.2% of cells). Overall, the results of cell cycle modifications revealed that the cellular effects of the **Ru-dca** complex are much more similar to the effects of *cisplatin*, while the **Os-dca** complex showed only a small effect on the cell cycle in the A2780 cells as compared to the negative control. Just for the comparative purposes we may show that similar human cancer cell cycle perturbations were recently reported for half-sandwich complexes [Ru(η^6^-*p*cym)(L^2^)Cl] and [Os(η^6^-*p*cym)(L^2^)Cl] containing 1-(4-methoxybenzyl)-4-phenyl-1*H*-1,2,3-triazole (HL^2^) [38].

### 2.7. Induction of Mitochondrial Membrane Potential Changes and Cytochrome c Release

In general, the cytotoxic effect of anticancer agents is often connected with the mitochondrial membrane potential alternation [39,40], as formerly described for various cytotoxic agents including the dca-containing Pt(IV) complex *mitaplatin* [4] or half-sandwich complexes similar to the herein reported complexes [13]. Further, it is generally accepted that the depolarization of mitochondrial membrane is usually connected with the Cytc release [39,41]. The released Cytc subsequently activates various proapoptotic signals in the cytosol (like formation of apoptosome), making Cytc an ultimate factor in the programmed cell death regulation. The Cytc release also most likely plays a crucial role in the mechanism of cancer cell resistance against the therapeutic action of anticancer drugs such as *cisplatin* [39,40]. Additionally, sodium dichloroacetate is well-known clinically studied mitochondria-targeting anticancer active small molecule [19]. This implied a possibility of the synergistic effect of the metal-based species and the dca ligand, both released from the studied complexes **Ru-dca** and **Os-dca**.

Concerning the **Ru-dca** and **Os-dca** complexes, these compounds induced only low mitochondrial membrane depolarization while 89.3% of cells, and 93.2% of cells, respectively, stayed intact. This result is also comparable with the conventional platinum-based drug *cisplatin* (88.8% of intact cells) (Table 3). 

**Figure 6:**
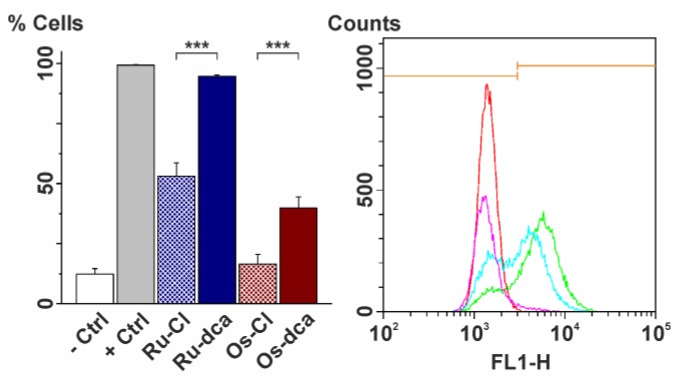
(**Left**) The results of the flow cytometry studies of cytochrome *c* release at the A2780 cells treated for 24 h by the IC_50_ concentrations of complexes **Ru-Cl** (dashed blue), **Ru-dca** (blue), **Os-Cl** (dashed red) and **Os-dca** (red); white = negative control (−Ctrl), grey = positive control (+Ctrl), *p* < 0.005 (***). (**Right**) The representative histogram of the flow cytometry studies of cytochrome *c* release, showing the fluorescence intensities in FITC-H channel in the A2780 cells treated by complexes **Ru-dca** (pink) and **Os-dca** (blue), given together with the negative (green) and positive (red) controls. Orange line segments show the edge between low and high fluorescence populations.

Although the above-mentioned results revealed that both the complexes caused only slight depolarization of mitochondrial membranes, the further flow-cytometry studies of cytochrome c (Cytc) cytosolic release [39,41], which is a causal pre-condition of apoptosome activation, were performed. The **Ru-dca** complex showed considerable induction of Cytc release from the mitochondria (94.6% of all cells were positive), while only the moderate Cytc release was induced by the **Os-dca** complex (39.9% of all cells were positive), as compared with the negative control (only 12.3% of all cells were Cytc positive) (see Figure 6). The effect of the **Ru-dca** complex almost reached the level of the positive control (Figure 6 and Table 4) and significantly exceeded both reference drugs *staurosporine* (47.1% of positive cells) and *cisplatin* (53.0% of positive cells). Furthermore, the induction of the Cytc release from the mitochondria in the A2780 cells was significantly higher for both dca-complexes as compared with their chlorido-analogues (see Table 4).

The studies of the ability of half-sandwich Ru(II) or Os(II) complexes to induce the Cytc release are quite rare in the literature. In particular, it has been reported that complexes RAPTA-C (Western blot analysis) [42] and [Os(η^6^-*p*cym)(L^2^)I]PF_6_ (flow cytometry studies) [43] induced the Cytc release in the treated human cancer cells; L^2^ = *N*,*N*-dimethyl-4-[(*E*)-pyridin-2-yl-diazenyl]-aniline.

A lack of correlation between the flow cytometry studies of the mitochondria membrane potential depletion and Cytc release induced by the studied complexes (Table 3 and Table 4) could be connected with various aspects of the Cytc release from mitochondria [44,45]. In other words, the Cytc release does not have to be necessarily a consequence of a Δψ_m_ loss, because it was reported that (a) the Cytc release occurs independently from the mitochondria membrane status [44], and (b) Cytc releases mitochondria through the multiple pathways [45].

## 3. Materials and Methods

### 3.1. Materials

The starting salts RuCl_3_∙*x*H_2_O and OsCl_3_∙*x*H_2_O were supplied by Precious Metals Online (University of Wollongong, Wollongong, Australia) and Sigma-Aldrich (Prague, Czech Republic), respectively. The chemicals 1-methyl-4-(propan-2-yl)cyclohexa-1,3-diene (α-terpinene), 4,7-diphenyl-1,10-phenanthroline (bathophenanthroline, bphen), dichloroacetic acid (Hdca), sodium hydroxide, silver(I) trifluoromethanesulfonate (AgOTf), ammonium hexafluorophosphate, l–cysteine (CySH), reduced glutathione (GSH), cytochrome c from bovine heart (Cytc) and hen egg-white lysozyme (HEWL) were purchased from VWR International (Stříbrná Skalice, Czech Republic) and Sigma-Aldrich (Prague, Czech Republic), while the solvents (methanol, diethyl ether, *n*-hexane, dichloromethane) were supplied by Litolab (Chudobín, Czech Republic). The deuterated solvents for the NMR experiments (DMSO-*d*_6_, CDCl_3_, MeOD-*d*_4_, D_2_O, DClO_4_ and KOD) were purchased from Sigma-Aldrich (Prague, Czech Republic).

[Ru(µ-Cl)(η^6^-pcym)Cl]_2_ and [Os(µ-Cl)(η^6^-pcym)Cl]_2_ were prepared in a microwave reactor Monowave 300 (Anton PaarGmbH, Graz, Austria), according to the reported protocols [46,47]. Silver(I) dichloroacetate, Ag(dca), was prepared as white solid from Hdca, which was neutralized by stoichiometric amount of 1 M NaOH (in methanol) providing Na(dca), which subsequently interacted in situ (15 min of stirring at ambient temperature in the dark) with 1 molar equiv. of AgOTf.

### 3.2. Synthesis

The formerly reported protocol for the preparation of complex **Ru-Cl** [20] was modified, as described in Supplementary Materials, and this modification was used also for new Os(II) analogue **Os-Cl**. Complexes **Ru-Cl** and **Os-Cl** (0.05 mmol) were dissolved in MeOH (15 mL) and an excess of Ag(dca) (0.25 mmol) was added to both solutions. The mixtures were stirred overnight without heating under the aluminum foil. Thereafter, the formed AgCl and unreacted Ag(dca) was removed, and NH_4_PF_6_ (0.5 mmol) was added to the filtrates, followed by the solvent evaporation combined with the addition of excess diethyl ether, until the solid formed. The crude products were recrystallized from the mixture of dichloromethane and *n*-hexane to give complexes [Ru(η^6^-*p*cym)(bphen)(dca)]PF_6_ (**Ru-dca**) and [Os(η^6^-*p*cym)(bphen)(dca)]PF_6_ (**Os-dca**) (Figure 1). The products were collected by filtration, washed (1 × 0.5 mL of MeOH and 3 × 1 mL of diethyl ether) and dried under vacuum. The yields were 45% (for **Ru-dca**) and 35% (for **Os-dca**), related to the starting Ru(II) and Os(II) dimers.

*Anal.* Calcd. for C_36_H_31_N_2_O_2_Cl_2_RuPF_6_ (**Ru-dca**): C, 51.44; H, 3.72; N, 3.33%; found: C, 51.06; H, 3.63; N, 3.14%. ^1^H NMR (DMSO-*d*_6_, ppm): *δ* 10.19 (d, *J* = 5.5 Hz, C2–H, 2H), 8.17 (d, *J* = 5.5 Hz, C3–H, 2H), 8.08 (s, C5–H, 2H), 7.67 (m, C9–H, C10–H, C11–H, 10H), 6.63 (d, *J* = 6.4 Hz, C23–H, 2H), 6.33 (d, *J* = 6.4 Hz, C22–H, 2H), 5.81 (s, C32–H, 1H), 2.66 (sep, *J* = 7.3 Hz, C25–H, 1H), 2.14 (s, C27–H, 3H), 1.01 (d, *J* = 7.3 Hz, C26–H, 6H). ^1^H NMR (CDCl_3_, ppm): *δ* 9.90 (d, *J* = 5.5 Hz, C2–H, 2H), 8.03 (s, C5–H, 2H), 7.98 (d, *J* = 5.5 Hz, C3–H, 2H), 7.59 (m, C9–H, C10–H, C11–H, 10H), 6.31 (d, *J* = 6.4 Hz, C23–H, 2H), 6.07 (d, *J* = 6.4 Hz, C22–H, 2H), 5.36 (s, C32–H, 1H), 2.70 (sep, *J* = 6.6 Hz, C25–H, 1H), 2.19 (s, C27–H, 3H), 1.16 (d, *J* = 6.4 Hz, C26–H, 6H). ^13^C NMR (DMSO-*d*_6_, ppm): *δ* 168.3 (C31), 156.4 (C2), 150.4 (C4), 146.2 (C7), 134.9 (C12), 130.0–127.5 (C6, C9, C10, C11), 125.4 (C5), 102.6 (C24), 102.4 (C21), 86.3 (C23), 83.3 (C22), 67.4 (C32), 30.6 (C25), 22.0 (C26), 18.1 (C27). ESI+ MS (methanol, *m*/*z*): 567.2 (calc. 567.1; 30%; {[Ru(*p*cym)(bphen)]–H}^+^), 695.1 (calc. 695.1; 100%; [Ru(*p*cym)(bphen)(dca)]^+^). IR (ATR, cm^–1^): 401, 489, 556, 636, 669, 702, 736, 765, 834, 927, 1000, 1020, 1029, 1055, 1077, 1191, 1231, 1325, 1403, 1420, 1426, 1445, 1470, 1494, 1518, 1537, 1562, 1598, 1659, 2846, 2923, 2965, 3057.

*Anal.* Calcd. for C_36_H_31_N_2_O_2_Cl_2_OsPF_6_ (**Os-dca**): C, 46.51; H, 3.36; N, 3.01%; found: C, 46.60; H, 3.20; N, 3.16%. ^1^H NMR (DMSO-*d*_6_, ppm): *δ* 10.12 (d, *J* = 5.5 Hz, C2–H, 2H), 8.15 (s, C5–H, 2H), 8.13 (d, *J* = 5.5 Hz, C3–H, 2H), 7.70 (m, C9–H, C10–H, C11–H, 10H), 6.88 (d, *J* = 5.5 Hz, C23–H, 2H), 6.53 (d, *J* = 5.5 Hz, C22–H, 2H), 5.86 (s, C32–H, 1H), 2.56 (sep, *J* = 6.4 Hz, C25–H, 1H), 2.23 (s, C27–H, 3H), 0.97 (d, *J* = 6.4 Hz, C26–H, 6H). ^1^H NMR (CDCl_3_, ppm): *δ* 9.84 (d, *J* = 5.5 Hz, C2–H, 2H), 8.08 (s, C5–H, 2H), 7.95 (d, *J* = 5.5 Hz, C3–H, 2H), 7.59 (m, C9–H, C10–H, C11–H, 10H), 6.56 (d, *J* = 5.8 Hz, C23–H, 2H), 6.28 (d, *J* = 5.8 Hz, C22–H, 2H), 5.33 (s, C32–H, 1H), 2.57 (sep, *J* = 6.8 Hz, C25–H, 1H), 2.26 (s, C27–H, 3H), 1.08 (d, *J* = 6.8 Hz, C26–H, 6H). ^13^C NMR (DMSO-*d*_6_, ppm): *δ* 168.1 (C31), 156.4 (C2), 150.8 (C4), 147.5 (C7), 134.7 (C12), 130.0–127.8 (C6, C9, C10, C11), 126.5 (C5), 101.6 (C21), 92.8 (C24), 85.1 (C23), 77.4 (C22), 66.8 (C32), 30.8 (C25), 22.4 (C26), 18.2 (C27). ESI+ MS (methanol, *m*/*z*): 657.3 (calc. 657.2; 45%; {[Os(*p*cym)(bphen)]–H}^+^), 785.1 (calc. 785.1; 100%; [Os(*p*cym)(bphen)(dca)]^+^). IR (ATR, cm^–1^): 401, 491, 557, 638, 669, 702, 735, 766, 835, 928, 971, 1000, 1021, 1028, 1054, 1134, 1232, 1268, 1322, 1407, 1420, 1427, 1445, 1470, 1495, 1519, 1558, 1601, 1628, 1660, 2871, 2927, 2967, 3065.

### 3.3. Methods

The ^1^H, ^13^C and ^1^H–^1^H gs-COSY spectra were recorded using a JEOL JNM-ECA 600II spectrometer (JEOL USA, Inc., Peabody, MA, USA) at 600.00 MHz (for ^1^H) and 150.86 MHz (for ^13^C) in the DMSO-*d*_6_ or CDCl_3_ solutions. The obtained ^1^H and ^13^C NMR spectra were calibrated against the residual signals (2.50 ppm for ^1^H NMR, 39.52 ppm for ^13^C NMR) of the used solvent [48]. The splitting of the ^1^H NMR signals is defined as s = singlet, d = doublet, sep = septet and m = multiplet. Mass spectrometry experiments were performed using a LCQ Fleet Ion Trap spectrometer (Thermo Scientific; Qual Browser software, version 2.0.7; Waltham, MA, USA) in the positive electrospray ionization mode (ESI+) in the methanolic solutions, and methanol/water (1:1, *v*/*v*) for interaction experiments, respectively. The protein mass spectra, measured in ESI+ mode, were deconvoluted by Promass for Xcalibur ver. 3.0, rev. 10 software (Novatia LLC, Newtown, PA, USA) to obtain the neutral mass spectra. Infrared spectra (400–4000 cm^–1^, ATR technique) were acquired by a Nexus 670 FT-IR (Thermo Nicolet; Waltham, MA, USA). Elemental analyses (C, H, N) were carried out by a Flash 2000 CHNS Elemental Analyzer (Thermo Scientific; Waltham, MA, USA). 

### 3.4. ^1^H NMR and ESI+ MS Studies of Hydrolytic Stability

The appropriate amount (for 600 µL of 1 mM solutions) of complexes **Ru-dca** and **Os-dca** were dissolved in MeOD-*d*_4_ (120 µL) and diluted by 480 µL of D_2_O. ^1^H NMR spectra were recorded on the fresh solutions (*t* = 0 h), and after 0.5, 1, 2, 3, 4, 6, 8 and 24 h of standing at ambient temperature. The obtained ^1^H NMR spectra were calibrated against the residual signal of D_2_O (4.85 ppm) [48]. For comparative purposes, the ^1^H NMR spectrum was also recorded for sodium dichloroacetate (Na(dca)) in 20% MeOD-*d*_4_/80% D_2_O.

Similar experiments were performed by ESI+ mass spectrometry for complexes **Ru-dca** and **Os-dca** (10 µM concentration) dissolved in the MeOH/H_2_O mixture (1:1, *v*/*v*). The spectra were recorded immediately after the sample preparation, and then after 1 h and after 24 h of standing at ambient temperature. 

### 3.5. Studies of Interactions with Biomolecules

The ESI+ MS studies of interactions of complexes **Ru-dca** and **Os-dca** with relevant biomolecules GSH and CySH were performed as follows: complexes (either **Ru-dca** or **Os-dca**) were dissolved in MeOH (500 µL, 10 µM final concentration) and 500 µL of the mixture of GSH (6 µM final concentration) and CySH (290 µM final concentration) in H_2_O was added. The obtained solutions were mixed properly and the ESI+ mass spectra were recorded immediately after preparation and after 1 and 24 h of standing at laboratory temperature.

Similarly, the interactions of complexes **Ru-dca** and **Os-dca** with the model protein lysozyme and cytochrome c were studied by means of ESI+ mass spectrometry. The samples contained the mixtures of either complex **Ru-dca** or **Os-dca** (at the 10 µM final concentration) with HEWL or Cytc (at the 3 µM final concentration) in the mixture of MeOH/H_2_O (1:1, *v/v*). The ESI+ mass spectra were recorded immediately after preparation and then after 1 and 24 h of standing of the mixtures at laboratory temperature. The specific experimental conditions were as follows: the sample solutions were introduced by HPLC (Dionex UltiMate 3000; Thermo Scientific; Waltham, MA, USA) in 50 µL spikes into the continual flow of methanol (0.2 mL/min flow rate), 5.3 kV spray voltage, 110 V and 275 °C capillary voltage and temperature. The obtained spectra were acquired in the range of 150–2000 *m*/*z* and the raw ESI+ spectra of proteins were deconvoluted by Promass for Xcalibur ver. 3.0, rev 10 software (Novatia LLC, Newtown, PA, USA) using the 0.1 Da mass step size. 

### 3.6. Cell Culture

The A2780 human ovarian carcinoma cell line (European Collection of Cell Cultures; Salisbury, UK) and primary culture of human hepatocytes (Hep, Biopredic Intl., Saint-Grégoire, France) were cultured according to the suppliers’ instructions, using RPMI-1640 medium supplemented with 10% of fetal calf serum, 1% of 2 mM glutamine and 1% penicillin/streptomycin (for the A2780 cells) and chemically defined medium consisting of a mixture of William’s E and Ham’s F-12 (1:1, *v*/*v*) (for the Hep cells), respectively. All cell lines were grown as adherent monolayers at 37 °C and 5% CO_2_ in a humidified atmosphere. 

#### 3.6.1. In Vitro Cytotoxicity

The 100 mM stock solutions of complexes **Ru-dca** and **Os-dca** were prepared by dissolving of appropriate amounts of these agents in 500 µL of DMF. *Cisplatin* and Hdca were involved in the testing for comparative purposes, and their stock solutions were of 80 mM and 10 M concentrations, respectively. 

The cultured A2780 cells were seeded to the 96-well culture plates, while the 96-well culture plates with the Hep cells were received from the supplier. The A2780 and Hep cells were pre-incubated in drug-free media at 37 °C for 24 h and then treated for the next 24 h at 37 °C with different concentrations of **Ru-dca**, **Os-dca**, *cisplatin,* and Hdca prepared from their stock solutions by dilution with RPMI-1640 medium (note: Hdca was studied only at the A2780 cells). After 24 h drug exposure, the supernatants were removed and the cells were washed with drug-free PBS followed by 72 h recovery in drug-free medium at 37 °C. The MTT assay (MTT = 3-(4,5-dimethylthiazol-2-yl)-2,5-diphenyltetrazolium bromide) was used to determine the cell viability. A concentration of the formazan formed from MTT was determined spectrophotometrically at 540 nm using an Infinite 200 PRO microplate reader (Tecan Group Ltd., Männedorf, Switzerland). 

The data were expressed as the percentage of viability, where 100% and 0% represents the treatments with negative (0.1% DMF in medium) and positive (1% Triton X-100) controls, respectively. The data from the cancer cells were acquired from three independent experiments (conducted in triplicate) using cells from different passages. The resulting IC_50_ values (µM) were calculated from the viability curves and the results are presented as arithmetic mean ± standard deviation (SD). 

#### 3.6.2. Cellular Accumulation 

The A2780 cells were seeded in 6-well culture plates (1 × 10^6^ cells per well) and incubated overnight (37 °C and 5% CO_2_ in a humidified incubator). Then the solutions, containing complexes **Ru-Cl**, **Os-Cl**, **Ru-dca** and **Os-dca** at the concentrations corresponding to IC_50_ from cytotoxicity testing, were added for the 24 h treatment, followed by washing with PBS (2 × 2 mL). The cells were harvested by trypsin, resuspended in PBS and centrifuged. The cell pellets were, immediately after the supernatant suction, digested in 500 µL of nitric acid (70 °C, overnight) to give the fully homogenized solutions. Then 4.5 mL of water was added and the final Ru and Os contents were determined by inductively coupled plasma mass spectrometry (ICP-MS; 7700× ICP-MS device, Agilent Technologies; Santa Clara, CA, USA) by external calibration using the 100 mg/L TraceCERT^®^ Transition metal mix 3 for ICP (Sigma Aldrich, Prague, Czech Republic). The obtained values were corrected for adsorption effects. The experiments were conducted in triplicate and the results are presented as arithmetic mean ± SD. 

### 3.7. Flow Cytometry Studies

For all the performed flow cytometry studies (cell cycle analysis, mitochondrial membrane potential assay and cytochrome c release), the A2780 cells were pre-incubated in the 6-well plates (1.0 × 10^6^ cells per well) for 24 h at 37 °C and 5% CO_2_ atmosphere. Complexes **Ru-Cl**, **Os-Cl**, **Ru-dca** and **Os-dca** (*cisplatin* was involved for comparative purposes) were added at the equipotent concentrations (equal to their IC_50_ concentrations from cytotoxicity testing). After 24 h exposure time, the cells were harvested using trypsin, washed twice with PBS and isolated by centrifugation. After that, the cells were stained as described below, and analyzed by a CytoFLEX Flow Cytometer (Beckman Coulter, Brea, CA, USA). In all cases, the untreated cells were used as a negative control, the experiments were conducted in triplicate (ca. 3 × 10^5^ cells per sample) and the obtained data were analyzed using CytExpert Software (Beckman Coulter, Brea, CA, USA).

#### 3.7.1. Cell Cycle Analysis

The washed cells were resuspended, stained by a propidium iodide (PI) solution supplemented with RNase A (25 °C, in the dark, 30 min) and analyzed by flow cytometry (excitation at 535 nm, emission detected at 617 nm).

#### 3.7.2. Mitochondrial Membrane Potential Assay

The A2780 cells were processed using the MITO-ID**^®^** Membrane potential detection kit (Enzo Life Sciences, Farmingdale, NY, USA), with the emission maxima detected at 525 nm (green dye) and 590 nm (orange dye). The positive control included the A2780 cells exposed to carbonyl cyanide 3-chlorophenylhydrazone (CCCP; 2 μM final concentration). 

#### 3.7.3. Cytochrome c Release

The cell staining procedure was performed according to the instructions supplied in the FlowCellect™ Cytochrome c Kit (Millipore, Burlington, MA, USA; Catalog No. FCCH100110) containing the Anti-IgG1-FITC Isotype Control (positive control) and Anti-Cytochrome c-FITC Antibody dye. For this experiment, the apoptosis inducer staurosporine (1 μg/mL concentration) was involved for comparative purposes as well.

### 3.8. Statistical Analysis

An ANOVA test was used for statistical analysis with the values of *p* < 0.05 (*), 0.01 (**) and 0.005 (***) considered to be statistically significant. QC Expert 3.2 Statistical software (TriloByte Ltd.; Pardubice—Staré Hradiště, Czech Republic) was used to perform the analyses.

## 4. Conclusions

Half-sandwich complexes [Ru(η^6^-*p*cym)(bphen)(dca)]PF_6_ (**Ru-dca**) and [Os(η^6^-*p*cym)(bphen)(dca)]PF_6_ (**Os-dca**) showed good in vitro cytotoxicity (IC_50_ = 3.5 μM, and 2.6 μM, respectively) against the A2780 human ovarian carcinoma cells, slightly exceeding the clinically-used platinum-based drug *cisplatin* (IC_50_ = 5.9 μM). Hydrolysis of the **Ru-dca** and **Os-dca** complexes is connected with the release of dichloroacetate (dca). The **Ru-dca** and **Os-dca** complexes do not interact with the model proteins cytochrome c (Cytc) and lysozyme. The results of the detailed flow cytometry studies (cell cycle perturbation, depolarization of mitochondrial membrane, Cytc release) were indicative for considerable differences in biological effects of the **Ru-dca** and **Os-dca** complexes at the used A2780 cells. The results point out the fact that the structural optimization of half-sandwich Ru(II) and Os(II) complexes represents a promising approach for the development of highly potent anticancer agents. 

## Figures and Tables

**Table 1:** Cellular accumulation of complexes **Ru-Cl**, **Ru-dca**, **Os-Cl** and **Os-dca** (calculated as the metal content) at the A2780 cells after the treatment for 24 h by the equipotent (IC_50_) concentrations. The data are given as arithmetic means from three independent experiments.

	ng/10^6^ Cells	fmol/10^6^ Cells
Ru-Cl	26.5 ± 0.7	262.2 ± 7.0
Ru-dca	37.0 ± 1.4	366.1 ± 14.0
Os-Cl	33.5 ± 1.4	174.3 ± 7.4
Os-dca	52.5 ± 1.2	273.1 ± 6.4

**Table 2:** The A2780 cell populations (%) in the cell cycle phases after the treatment for 24 h by the IC_50_ concentrations of complexes **Ru-Cl**, **Ru-dca**, **Os-Cl** and **Os-dca**, given together with negative control (untreated cells) and *cisplatin* (for comparative purposes). The data are given as arithmetic means from three independent experiments.

	Sub-G_1_	G_0_/G_1_	S	G_2_/M
Ru-Cl	0.7 ± 0.2	62.4 ± 1.8	13.1 ± 2.8	23.2 ± 2.9
Ru-dca	16.8 ± 1.9	37.0 ± 2.7	24.3 ± 3.3	20.9 ± 1.1
Os-Cl	0.8 ± 0.3	59.0 ± 2.3	14.5 ± 0.9	25.1 ± 1.5
Os-dca	0.7 ± 0.2	62.9 ± 1.3	13.6 ± 4.7	22.2 ± 4.5
*Cisplatin*	8.0 ± 0.9	26.1 ± 2.1	30.9 ± 4.9	34.5 ± 3.5
Control	0.8 ± 0.3	69.2 ± 2.2	15.7 ± 1.4	13.9 ± 1.1

**Table 3:** The results (given as % cell populations) of flow cytometry studies of the mitochondrial membrane potential changes, studied at the A2780 cells using the 24 h treatment by the IC_50_ concentrations of complexes **Ru-Cl**, **Ru-dca**, **Os-Cl** and **Os-dca** (and *cisplatin* for comparative purposes). The untreated A2780 cells were employed as the negative control, while the CCCP-treated cells represent the positive control. The data are given as arithmetic means ± SD from three independent experiments.

	% of Cells Showing High PE-Channel Fluorescence
Ru-Cl	94.0 ± 1.4
Ru-dca	89.3 ± 1.4
Os-Cl	95.2 ± 0.7
Os-dca	93.2 ± 2.3
*Cisplatin*	88.8 ± 2.6
Positive control	38.6 ± 1.7
Negative control	99.1 ± 0.3

**Table 4:** The results (given as % cell populations) of the cytochrome *c* release studied by flow cytometry at the A2780 cells treated for 24 h by the IC_50_ concentrations of complexes **Ru-Cl**, **Ru-dca**, **Os-Cl** and **Os-dca** (*cisplatin* and *staurosporine* were used for comparative purposes). The untreated A2780 cells were employed as the negative control, while the cells applied with the Anti-IgG1-FITC Isotype represented the positive control. The data are given as arithmetic means ± SD from three independent experiments.

	% of Cells Showing Shift in FITC-Channel Fluorescence
Ru-Cl	53.0 ± 5.7
Ru-dca	94.6 ± 0.6
Os-Cl	16.5 ± 4.1
Os-dca	39.9 ± 4.6
Staurosporine	47.1 ± 6.1
*Cisplatin*	53.0 ± 6.0
Positive control	99.3 ± 0.1
Negative control	12.3 ± 2.3

## Supplementary Materials

Supplementary materials are available online: synthesis of **Ru-Cl** and **Os-Cl**, ^1^H NMR spectra of **Ru-dca** and **Os-dca** dissolved in CDCl_3_ Figure S1), ^1^H NMR (Figure S2) and ESI+ MS (Figure S3) studies of solution behavior in water-containing media, ESI+ MS (Figure S4) studies of the interaction of **Ru-dca** and **Os-dca** with GSH and CySH and mass spectra of cytochrome c and its mixture with **Ru-dca** (Figure S5).
