# Supplementary material for: Half-Sandwich Ru(II) and Os(II) Bathophenanthroline Complexes Containing a Releasable Dichloroacetato Ligand
Source: Molecules. 2018 Feb 14;23(2):420. doi: 10.3390/molecules23020420 (PMC6017048; doi:10.3390/molecules23020420)
Supplement: Supplementary file 1 [file molecules-23-00420-s001.pdf]

Supplementary Materials

for

Half-Sandwich Ru(II) and Os(II) Bathophenanthroline Complexes Containing a Releasable Dichloroacetato Ligand

Pavel Štarha [1], Zdeněk Trávníček [1,*], Ján Vančo [1] and Zdeněk Dvořák [2]

[1] Department of Inorganic Chemistry & Regional Centre of Advanced Technologies and Materials, Faculty of Science, Palacký University in Olomouc, 17. listopadu 12, 771 46 Olomouc, Czech Republic

[2] Department of Cell Biology and Genetics & Regional Centre of Advanced Technologies and Materials, Faculty of Science, Palacký University in Olomouc, Šlechtitelů 27, 783 71 Olomouc, Czech Republic

Synthesis

The formerly reported protocol for the preparation of complex **Ru-Cl** [1] was modified, as described below, and this modification was used also for the Os(II) analogue **Os-Cl**. The starting dimer [M(μ-Cl)(η^6-*p*cym)Cl]$_2$ (0.10 mmol; M = Ru or Os) reacted with an excess (0.15 mmol) of bphen in 5 mL of MeOH in a microwave reaction system (100 °C, 1 min). The obtained solutions were cooled to ambient temperature, and an excess of NH_4PF_6 (3.0 mmol) was added. The solvent volume was reduced after 15 min of stirring at ambient temperature, until the solid formed. The obtained chlorido complexes [Ru(η^6-*p*cym)(bphen)Cl]PF_6 (**Ru-Cl**) and [Os(η^6-*p*cym)(bphen)Cl]PF_6 (**Os-Cl**) were collected by filtration, washed (1 × 0.5 mL of MeOH and 3 × 1 mL of diethyl ether) and dried under vacuum.

Anal. Calcd. for $C_{34}H_{30}N_2ClRuPF_6$ (**Ru-Cl**): C, 54.59; H, 4.04; N, 3.74%; found: C, 54.46; H, 3.92; N, 3.59%. ^{1}H NMR (DMSO-*d*$_6$, ppm): δ 10.01 (d, *J* = 5.5 Hz, C2–H, 2H), 8.16 (d, *J* = 5.5 Hz, C3–H, 2H), 8.13 (s, C5–H, 2H), 7.65 (m, C9–H, C10–H, C11–H, 10H), 6.40 (d, *J* = 6.4 Hz, C23–H, 2H), 6.20 (d, *J* = 6.4 Hz, C22–H, 2H), 2.72 (sep, *J* = 6.4 Hz, C25–H, 1H), 2.19 (s, C27–H, 3H), 1.02 (d, *J* = 6.4 Hz, C26–H, 6H). ^{13}C NMR (DMSO-*d*$_6$, ppm): δ 155.8 (C2), 150.0 (C4), 145.9 (C7), 134.9 (C12), 130.0–127.6 (C6, C9, C10, C11), 125.5 (C5), 104.8 (C21), 102.2 (C24), 85.9 (C23), 84.5 (C22), 30.5 (C25), 21.8 (C26), 18.2 (C27). ESI+ MS (methanol, *m*/*z*): 603.1 (calc. 603.1; 100%; [Ru(*p*cym)(bphen)Cl]$^+$). IR (ATR, cm^{-1}): 408, 461, 490, 515, 556, 636, 670, 699, 736, 764, 836, 925, 999, 1030, 1079, 1160, 1229, 1298, 1403, 1444, 1469, 1494, 1517, 1559, 1598, 1621, 2872, 2932, 2968, 3030, 3050, 3090.

Anal. Calcd. for $C_{34}H_{30}N_2ClOsPF_6$ (**Os-Cl**): C, 48.77; H, 3.61; N, 3.35%; found: C, 48.80; H, 3.46; N, 3.27%. ^{1}H NMR (DMSO-*d*$_6$, ppm): δ 9.95 (d, *J* = 5.5 Hz, C2–H, 2H), 8.19 (s, C5–H, 2H), 8.13 (d, *J* = 5.5 Hz, C3–H, 2H), 7.70 (m, C9–H, C10–H, C11–H, 10H), 6.62 (d, *J* = 5.9 Hz, C23–H, 2H), 6.37 (d, *J* = 5.9 Hz, C22–H, 2H), 2.57 (m, C25–H, 1H), 2.25 (s, C27–H, 3H), 0.95 (d, *J* = 6.4 Hz, C26–H, 6H). ^{13}C NMR (DMSO-*d*$_6$, ppm): δ 155.6 (C2), 150.1 (C4), 147.0 (C7), 134.8 (C12), 130.2–127.8 (C6, C9, C10, C11), 125.9 (C5), 95.5 (C21), 94.9 (C24), 77.5 (C23), 75.0 (C22), 30.6 (C25), 22.1 (C26), 18.1 (C27). ESI+ MS (methanol, *m*/*z*): 693.2 (calc. 693.2; 100%; [Os(*p*cym)(bphen)Cl]$^+$). IR (ATR, cm^{-1}): 407, 465, 489, 555, 637, 669, 699, 734, 764, 833, 927, 999, 1029, 1055, 1079, 1154, 1185, 1230, 1273, 1300, 1404, 1444, 1468, 1493, 1516, 1557, 1600, 1624, 2876, 2933, 2967, 3029, 3052, 3092.

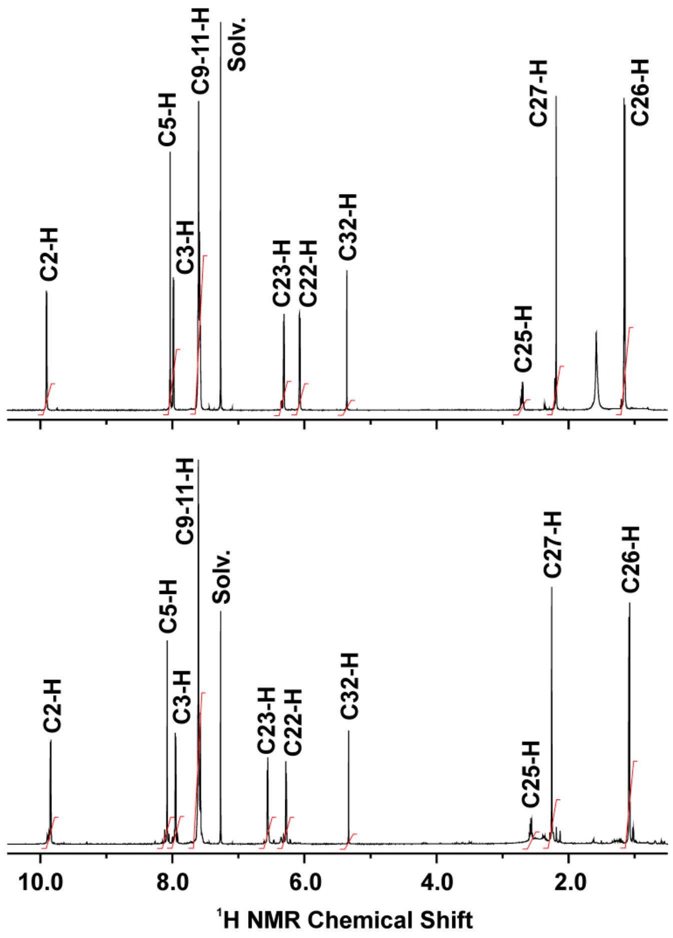

Figure S1. ^1H NMR spectra ($CDCl_3$ solutions) of complexes **Ru-dca** (*bottom*) and **Os-dca** (*top*) given together with the assignment of the detected signals.

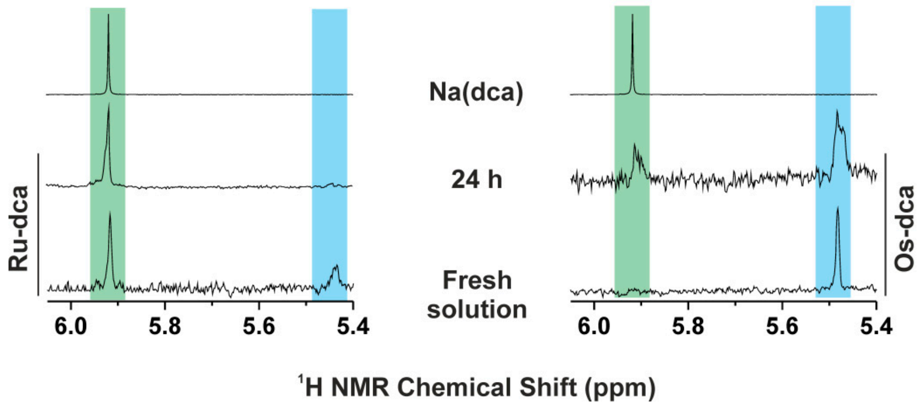

Figure S2. The selected results of the time-dependent ^{1}H NMR studies of the progress of the dca ligand release of complexes **Ru-dca** and **Os-dca** dissolved in 20% MeOD-d_4/80% D_2O. The signals of the C32–H hydrogen atom of the coordinated and released dca is depicted in blue, and green, respectively.

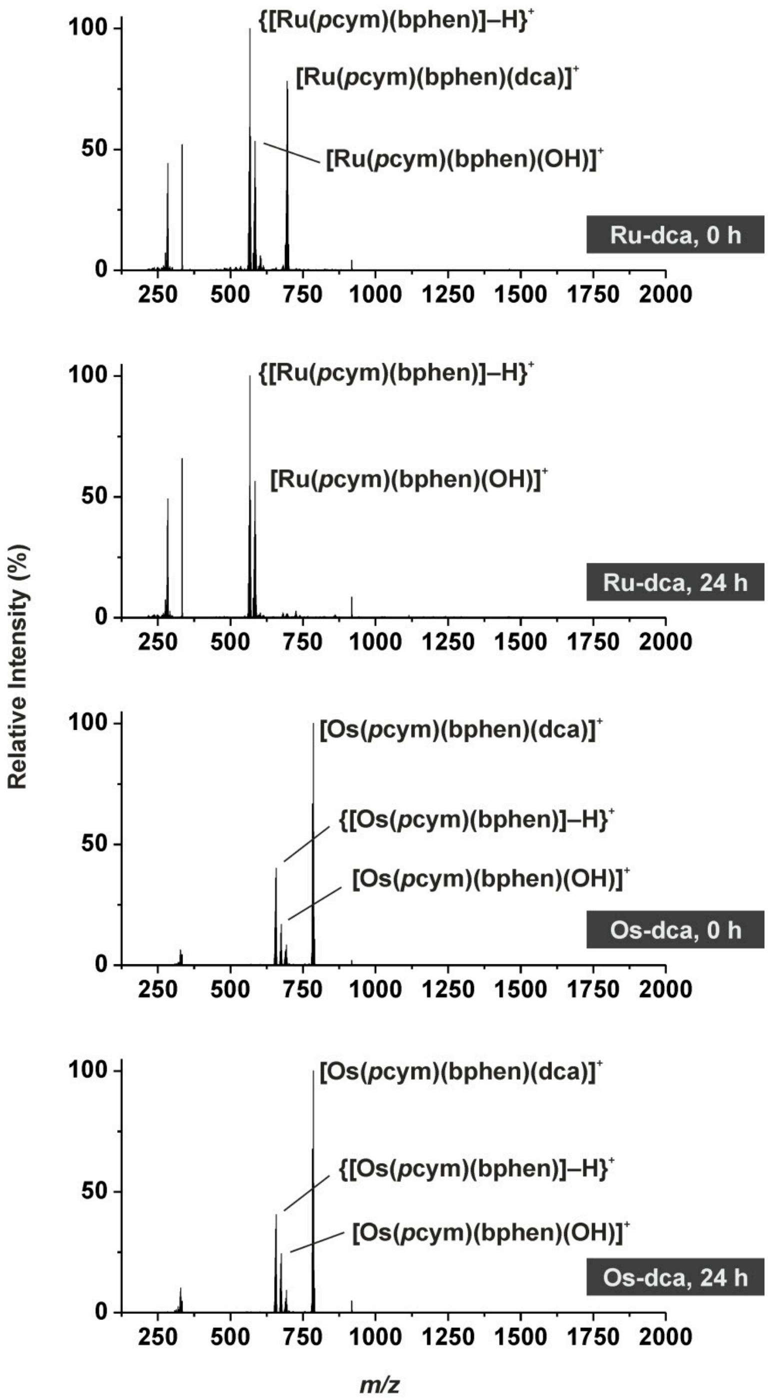

Figure S3. ESI+ mass spectra of complexes **Ru-dca** and **Os-dca** dissolved in methanol/water (1:1, *v/v*) obtained at various time points.

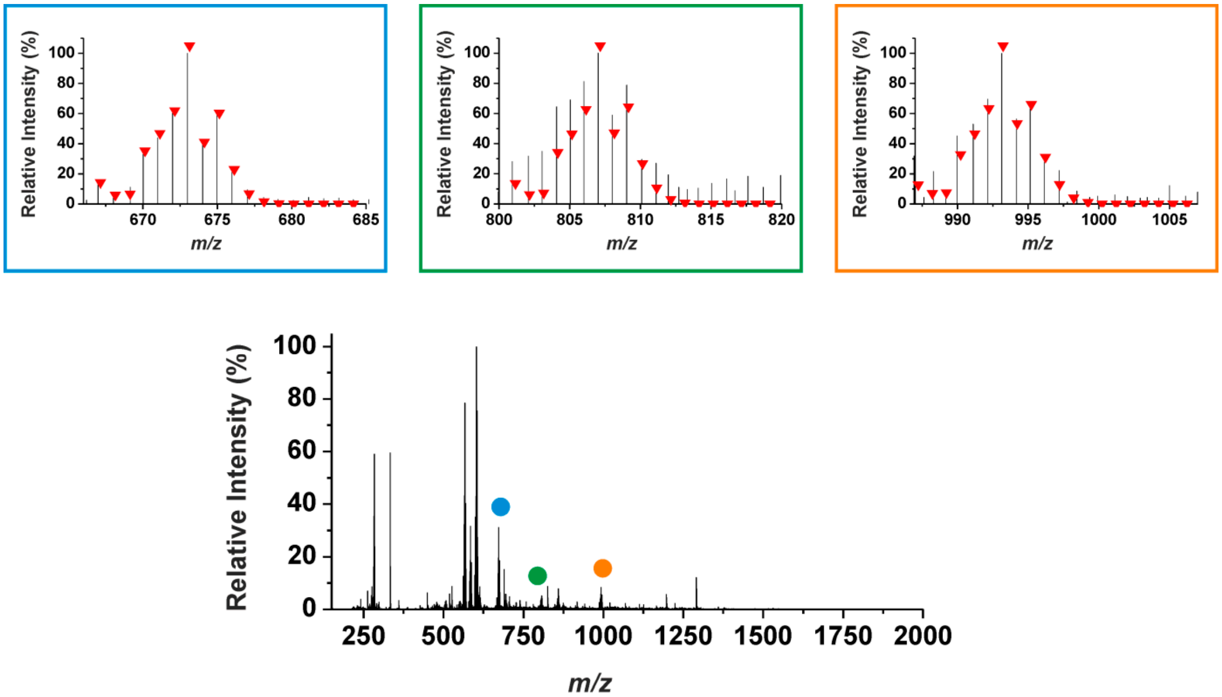

Figure S4. ESI+ mass spectra of the mixture of complex **Ru-dca** and GSH (6 µM final concentration) and CySH (290 µM final concentration) in methanol/water (1:1, *v/v*) recorded after 24 h of standing at ambient temperature. The peaks of the adducts of $\{[Ru(p\text{cym})(\text{bphen})]{-}H\}^+$ with either two CyS (or one cystine (CySSCy); 807.2 *m/z*; green sphere) or with CyS and GS (or their disulfide CySSG; 993.2 *m/z*; orange sphere) and the adduct of $\{[Ru(p\text{cym})(\text{bphen})]{+}(HL^3){-}H\}^+$ with deaminated cysteine (*i.e.*, 3-sulfanylpropanoic acid; HL^3; 673.0 *m/z*; blue sphere) are given in detail together with the calculated isotopic distribution (red triangles).

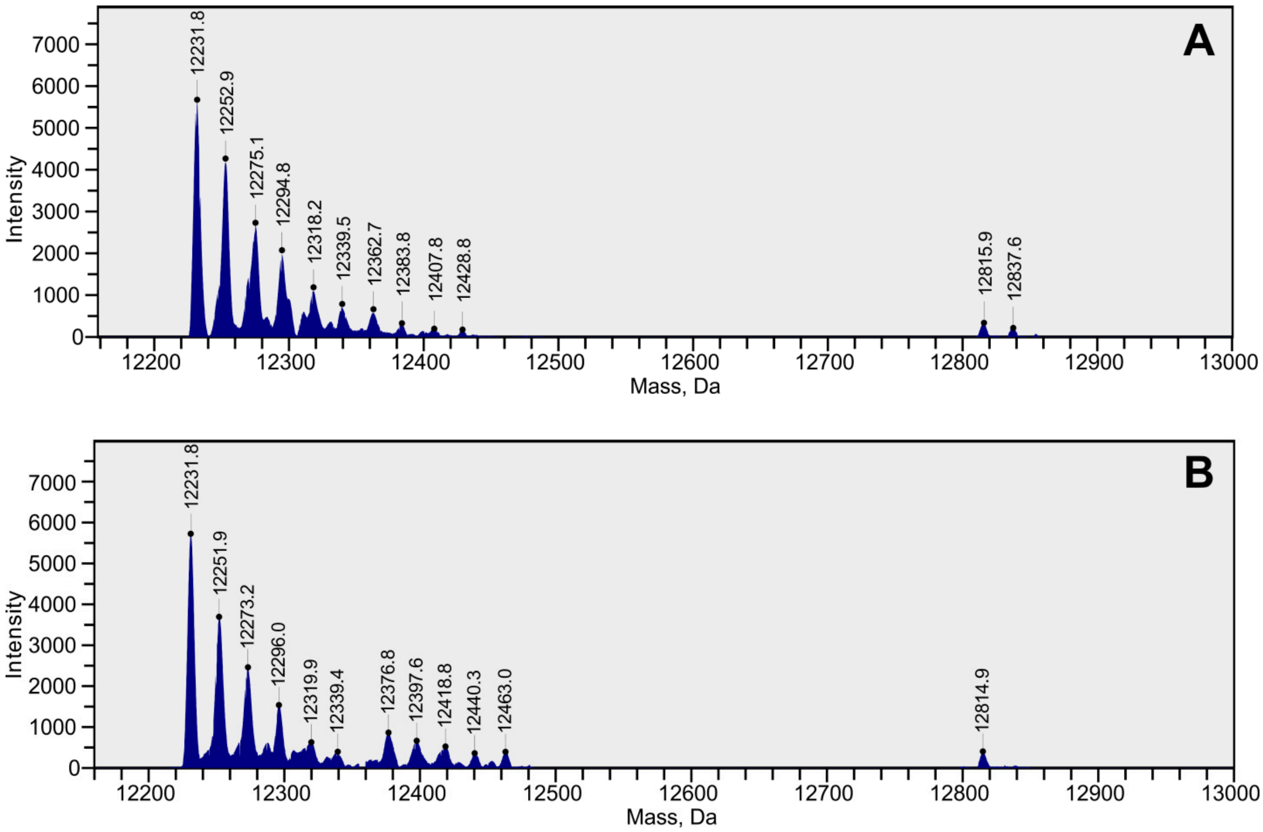

Figure S5. Deconvoluted neutral mass spectra of cytochrome c (Cytc; A) and its mixture (3 μM final concentration) with complex **Ru-dca** (10 μM final concentration) (B), both dissolved in MeOH/H_2O (1:1, *v/v*). The mass spectra were recorded after 24 h of standing at room temperature and show the formation of a minor adduct of Cytc with PF_6^-, characterized by the mass difference of 145 Da (difference between the main peak of Cytc at 12231.8 Da and main peak of the {Cytc–PF_6} adduct at 12376.8 Da).

References

[1] Betanzos-Lara, S.; Novakova, O.; Deeth, R.J.; Pizarro, A.M.; Clarkson, G.J. Liskova, B.; Brabec, V.; Sadler, P.J.; Habtemariam, A. Bipyrimidine ruthenium(II) arene complexes: structure, reactivity and cytotoxicity. *J. Biol. Inorg. Chem.* **2012**, *17*, 1033–1051.
